# Symptoms of chronic rhinosinusitis with and without nasal polyps

**DOI:** 10.1186/2045-7022-3-S2-O2

**Published:** 2013-07-16

**Authors:** Dirk Dietz de Loos, Claire Hopkins, Wytske Fokkens

**Affiliations:** 1 Academic Medical Centre, Otorhinolaryngology, Amsterdam, the Netherlands; 2 Guy’s Hospital, Otorhinolaryngology, London, UK

## Background

According to EPOS, chronic rhinosinusitis with and without nasal polyps diagnoses are defined by clinical criteria, supported with endoscopy. We want to know if it is possible to make an accurate distinction between patients with and without nasal polyps based on a limited number of clinical criteria, collected by health-related quality of life questionnaires. A validated instrument that can differentiate patients with chronic rhinosinusitis with and without nasal polyps could be used in epidemiologic research.

## Methods

We collected RSOM-31 (Rhinosinusitis Outcome Measurement) questionnaires from CRS patients with and without nasal polyps and compared mean total RSOM-31 scores, mean domain scores, mean symptoms scores, and percentages of patients reporting symptoms per diagnosis based on endoscopy and CT scan. Furthermore a prediction model was obtained by multivariable regression analysis, to define individual risk for nasal polyps in daily practice.

## Results

RSOM-31 Questionnaires were collected from 234 patients. Although total RSOM-31 score was similar and symptomatology considerably overlapping, patients with CRSwNP (with nasal polyps) scored significantly higher and more often on nasal symptoms as 'Rhinorrhoea' and 'Decreased sense of taste or smell'. Patients with CRSsNP (without nasal polyps) significantly scored more often and higher on 'facial pain' and 'ear pain'. A prediction model containing patient characteristics and the RSOM-31 items 'Rhinorrhoea', 'Decreased sense of taste or smell', 'facial pain', 'ear pain' and 'Inconvenience of always having to carry tissues around' gives a sensitivity of 81% and specificity of 61% on the outcome 'nasal polyps'.

## Conclusion

Although there are significant differences scored on several symptoms, there is considerable overlap of many symptoms and it remains difficult to distinguish between CRSwNP and CRSsNP based on clinical impression alone. We constructed a prediction model that makes a distinction between patients with and without nasal polyps with a certain sensitivity and specificity. This instrument could be used for epidemiologic research on chronic rhinosinusitis.

